# Exploration of Superior Modality: Safety and Efficacy of Hypofractioned Image-Guided Intensity Modulated Radiation Therapy in Patients with Unresectable but Confined Intrahepatic Hepatocellular Carcinoma

**DOI:** 10.1155/2017/6267981

**Published:** 2017-10-01

**Authors:** Tao Jiang, Zhao-Chong Zeng, Ping Yang, Yong Hu

**Affiliations:** Department of Radiation Oncology, Zhongshan Hospital, Fudan University, Shanghai 200032, China

## Abstract

**Purpose:**

To evaluate the efficacy and safety of hypofractioned image-guided intensity modulated radiation therapy (IG-IMRT) for unresectable but confined intrahepatic hepatocellular carcinoma in comparison with conventional 3-dimensional conformal radiotherapy (3D-CRT).

**Methods:**

Ninety patients with unresectable but confined intrahepatic hepatocellular carcinoma without distant metastasis and tumor thrombosis received external beam radiation therapy. Of these patients, 45 received IG-IMRT and 45 received 3D-CRT. The IG-IMRT design delivered a median total hypofractionated dose of 54 Gy (2.2–5.5 Gy/fx), and 3D-CRT delivered a median total dose of 54 Gy with a conventional fraction (2.0 Gy/fx). The clinical response, overall survival, and side effects were analyzed.

**Results:**

The IG-IMRT group showed significantly higher 1-year survival (93.3 versus 77.8%) and 2-year survival (73.3 versus 51.1%) and longer median survival (44.7 versus 24.0 months) than the 3D-CRT group. Multivariate analysis indicated that the patients with intrahepatic tumors smaller than 8 cm, prior TACE before RT, and IG-IMRT would have a survival benefit. There were no significant differences in the rates of side effects between the two groups.

**Conclusion:**

Hypofractioned IG-IMRT could improve the therapeutic response and confer a potential survival of patients with unresectable but confined intrahepatic hepatocellular carcinoma compared to 3D-CRT with acceptable toxicity.

## 1. Introduction

Worldwide, liver cancer in men is the fifth most frequently diagnosed cancer but the second most frequent cause of cancer-related death. In women, it is the seventh most commonly diagnosed cancer and the sixth leading cause of cancer-related death [1]. An estimated 748,300 new liver cancer cases and 695,900 liver cancer-related deaths occurred worldwide in 2008. Half of these cases and deaths were estimated to occur in China [2]. Among primary liver cancers, hepatocellular carcinoma (HCC) represents the major histological subtype, accounting for 70% to 85% of the total liver cancer burden [3]. Several modalities have been used for the treatment of HCC, including surgical resection, liver transplantation, transarterial chemoembolization (TACE), radiofrequency ablation (RFA), percutaneous ethanol injection (PEI), and radiotherapy. Because of poor tolerance of the entire liver to radiation, conventional external beam radiotherapy (EBRT) has been limited as a therapeutic option for the treatment of HCC [4]. However, this limitation has been overcome by new radiotherapy modalities, especially image-guided radiotherapy (IGRT) that delivers high-dose radiation. Several studies have shown that the administration of a higher radiation dose results in higher survival rates for HCC patients [5]. Helical tomotherapy is a dedicated intensity modulated radiation therapy (IMRT) system with on-board imaging capability with megavoltage computerized tomography (MVCT) that differs from conventional treatment units, and the imaging capacity conferred by the CT component allows targeted regions to be visualized prior to, during, and immediately after each treatment. The MVCT images supplant port films to provide unprecedented anatomical detail. Additional benefits include better dose coverage for target volumes and the sparing of nearby organs [6, 7].

In our study, we compared the oncologic effects of IG-IMRT to 3D-CRT to assess the efficacy and safety of hypofractioned IG-IMRT by helical tomotherapy for unresectable but confined intrahepatic hepatocellular carcinoma.

## 2. Methods

### 2.1. Patients

This study retrospectively reviewed 90 patients with unresectable but confined intrahepatic hepatocellular carcinoma without lymph node metastasis, vascular invasion, and distant metastasis who received EBRT at our institution between April 2009 and December 2014. The diagnosis of hepatocellular carcinoma was based on the diagnosis criteria of the American Association for the Study of Liver Diseases (AASLD) [8, 9]. Abdominal enhanced computed tomography (CT) and/or magnetic resonance imaging (MRI), chest CT, and radionuclide bone scan were evaluated before radiotherapy. Child-Pugh classification C and/or extrahepatic metastases were excluded from RT. All of the enrolled patients had unresectable intrahepatic tumors that were mainly due to poor liver function (Child-Pugh B), tumor location, inadequate future liver remnant for huge intrahepatic tumor, more than 3 lesions in different hepatic lobes or segments, poor general health, or refusal to accept the surgery, which had been diagnosed by surgeons as unsuitable cases for surgery, and 21 patients had contraindications for TACE.

This retrospective single-institution study was approved by local ethics review board (ID: 2011235).

### 2.2. Treatment

The patients who received RT in our research were the cases with incomplete TACE. None of the cases underwent radiotherapy for the newly developed lesions. 3D-CRT or IG-IMRT was performed based on the patient's choice and the status of the disease. IG-IMRT has a dose distribution advantage over multiple lesions or adjacent gastrointestinal tumors and is recommended for such patients. Patients that received radiotherapy were in the supine position with arms raised and vacuum pad fixed posture, and we used abdominal compression (AC) techniques as part of a fixed position to minimize the movement of liver. The AC was applied to the subxiphoid area under patient's maximum tolerability [10]. Simulation CT was performed with an abdominal 4D-CT enhanced scan. Two additional series of CT scans were obtained during inspiration and expiration to track the motion of the tumors and other internal organs. Fusion with MRI or PET/CT images to determine the range of the target region was also applied when necessary. The visible lesions were contoured as a gross tumor volume (GTV). GTV was expanded by 5 mm to create a clinical target volume (CTV), and internal target volume (ITV) was determined by the activity of intrahepatic lesions during the respiratory cycle. The planning target volume (PTV) added a margin of 5 mm to the ITV to compensate for daily setup errors and target motion [11, 12].

There were 45 patients who received IG-IMRT by helical tomotherapy, and 45 received 3D-CRT. The median fraction dose of IG-IMRT was 3.2 Gy (2.2–5.5 Gy/fx), and the median total hypofractionated dose was 54 Gy (range, 35–68 Gy). The 3D-CRT was designed to deliver a median total dose of 54 Gy (range, 46–70 Gy) with a conventional fraction (2.0 Gy/fx). Radiotherapy was delivered once per day, 5 times a week. Both 3D-CRT and IG-IMRT were performed with 95% of the goal dose to cover 95% of the PTV. The prescription dose of radiotherapy was determined mainly according to the mean dose to the liver, which was limited to 23 Gy and was also limited by the tolerance dose of the gastrointestinal tract. Organs at risk (OARs) were under the tolerance dose, including the liver, kidneys, stomach, small intestine, and spinal cord.

The treatment applied after radiotherapy was variable. In the IG-IMRT group, 19 patients (42.2%) underwent TACE, 6 patients (13.3%) underwent liver cancer resection, 1 patient (2.2%) underwent liver transplantation, and 1 patient (2.2%) underwent radiofrequency ablation. In the 3D-CRT group, 28 patients (62.2%) underwent TACE, 3 patients (6.67%) underwent liver cancer resection, 1 patient (2.2%) underwent liver transplantation, and 2 patients (4.4%) underwent radiofrequency ablation.

### 2.3. Response Evaluation and Follow-Up

The patients were evaluated weekly during treatment, and this included physical examination, complete blood counts, and liver function tests. After treatment, the evaluation was performed monthly. The responses to therapy were confirmed by CT scan or MRI during follow-up, 1.5–2 months after the completion of EBRT. Clinical and radiological follow-up was performed every 3 months during the first 24 months following treatment and every 6 months thereafter. All images were reviewed by an independent radiologist who classified responses according to the revised Response Evaluation Criteria in Solid Tumors (version 1.1) [13]. A Complete Response (CR) was defined as a complete disappearance of all target lesions. A Partial Response (PR) was defined as at least a 30% decrease in the sum of diameters of target lesions. Progressive Disease (PD) was defined as at least a 20% increase in the sum of diameters of target lesions, and Stable Disease (SD) was defined as neither a sufficient shrinkage to qualify for PR nor sufficient increase to qualify for PD. The objective response rate (ORR) was defined as the percentage of patients with CR+PR, and the disease control rate (DCR) was defined as the percentage of patients with CR+PR+SD. Toxicity was classified by the National Cancer Institute Common Terminology Criteria for Adverse Events (NCI-CTCAE) v4.0 [14].

### 2.4. Statistical Analysis

Overall survival (OS) was calculated from the day of first treatment of the primary tumor. Progression-free survival (PFS) was defined as the time from radiotherapy start date to the date of target lesions progression, relapse, patient death, or the last contact. The chi-square test and an independent samples *t*-test were used to compare the baseline characteristics, dose distributions, responses to treatment, and treatment related toxicities between the two groups (IG-IMRT versus. 3D-CRT). OS and PFS were estimated using the Kaplan–Meier survival analyses, and the log-rank test was used for statistical comparison of the survival curves. Multivariate analysis of survival was carried out with Cox's regression model, and all variables were entered in a single step using backward stepwise regression. We considered a *P* value less than 0.05 as statistically significant. SPSS version 22.0.0 was used for all analyses.

## 3. Results

### 3.1. Patient Characteristics

The baseline demographic, clinical, and laboratory characteristics are shown in Table 1. No significant differences were seen between the two groups for all patient characteristics except Karnofsky Performance Score (KPS). The KPS of all patients was 80 or above, and the scores were higher in the 3D-CRT than in the IG-IMRT group (*P* = 0.035).

Most of the patients received TACE before radiotherapy. Chemotherapeutic agents and embolization agents were selected according to the tumor location and size. The percentages of patients that received TACE were similar between the IG-IMRT group and the 3D-CRT group, and the average TACE frequency was 2.4 and 2.9, respectively. However, neither the percentage nor frequency of TACE administration before RT was significantly different between the groups.

### 3.2. Dose Distribution

**Figure 1:**
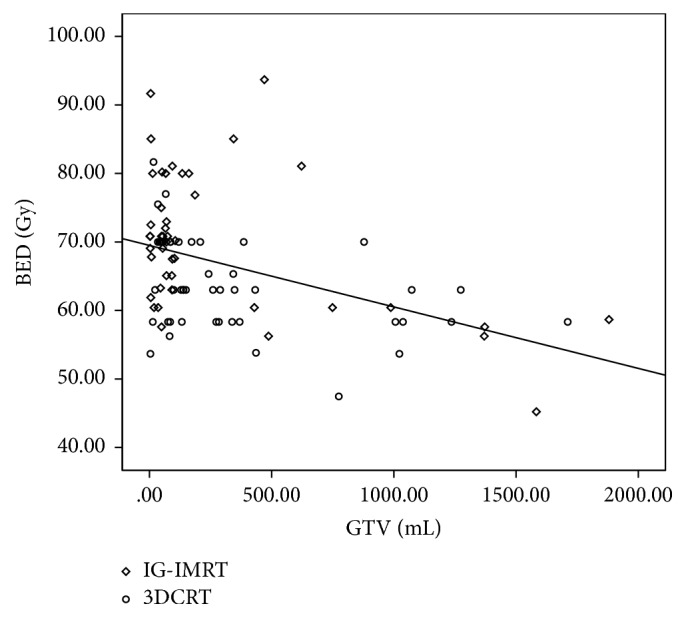
Correlation between GTV and BED. Correlation analysis showed that the prescription dose was negatively correlated with GTV. The correlation coefficient was −0.431 (*P* < 0.001).

Because of the performance status of patients, size and location of the tumor, and the limits of the OARs, the prescription dose and fraction of radiotherapy were different. Correlation analysis showed that the prescription dose was negatively correlated with GTV, which was determined by tumor size. The correlation coefficient was −0.431 (*P* < 0.001, Figure 1).

To make the radiation doses comparable, the total dose was converted to the biologically effective dose (BED) using an L-Q model with an HCC *α*/*β* ratio of 12 Gy [15]. The BED of IG-IMRT was significantly different than that of 3D-CRT (*P* = 0.004), and the radiotherapy fractions in the IG-IMRT group were significantly less than that of the 3D-CRT group (*P* = 0.001). The percentage of whole liver covered by at least 5 Gy (V5) was significantly higher in IG-IMRT plans than in 3D-CRT plans (*P* = 0.001); however, V10, V20, and V30 of the whole liver and the mean dose to normal liver (MDTNL) showed no significant differences, as shown in Table 2.

### 3.3. Response to Treatment

In comparing the response to IG-IMRT with that to 3D-CRT, the CR rate was 8.9% (4/45) versus 4.4% (2/45), the PR rate was 48.9% (22/45) versus 28.9% (13/45), the percentage of those with SD was 37.8% (17/45) versus 55.6% (25/45), and the percentage of those with PD was 4.4% (2/45) versus 11.1% (5/45), respectively. The ORR (CR+PR) was significantly higher in the IG-IMRT group (*P* = 0.020), at 57.8% (26/45) versus 33.3% (15/45) in the 3D-CRT group. The DCR (CR+PR+SD) was similar (*P* = 0.238), at 95.6% (43/45) in the IG-IMRT group versus 88.9% (40/45) in the 3D-CRT group.

After radiotherapy, there were 11 patients having Child-Pugh scores decreased in IG-IMRT group and 12 patients in 3DCRT group, 4 patients, and 3 patients improved from Child-Pugh B to Child-Pugh A in IG-IMRT and 3-DCRT group, respectively. The tumors in 7 patients shrunk to less than 5 cm in both groups, which might convert patients to resectable cases.

### 3.4. Survival Analysis

**Figure 2:**
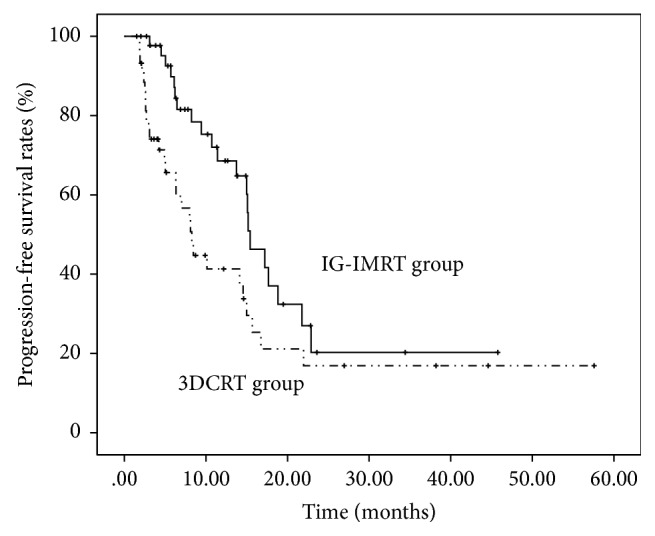
PFS rates of target lesions according to the modality of radiotherapy. The IG-IMRT group had a significantly longer median progression-free survival time of target lesions (*P* = 0.021). The median progression-free survival in the IG-IMRT group and the 3D-CRT group was 15.41 ± 1.51 months and 8.26 ± 1.00 months, respectively. The 1- and 2-year progression-free rates were 68.5% and 20.3% in the IG-IMRT group and 41.4% and 16.9% in the 3D-CRT group, respectively.

As to survival analysis, the median follow-up of all patients was 44.8 months, that for patients in HT group was 51.9 months (4.5–75.4 months), and that for patients in 3D-CRT group was 34.6 months (2.1–56.2 months), respectively. Target lesions in the radiation field were followed to evaluate progression-free survival in the two groups. 21 patients in the IG-IMRT group and 28 patients in the 3D-CRT group had target lesion progression or relapse. The median progression-free survival in the IG-IMRT group and the 3D-CRT group was 15.41 ± 1.51 months and 8.26 ± 1.00 months, respectively. Furthermore, the IG-IMRT group had a significantly longer median progression-free survival time (*P* = 0.021, Figure 2).

**Figure 3:**
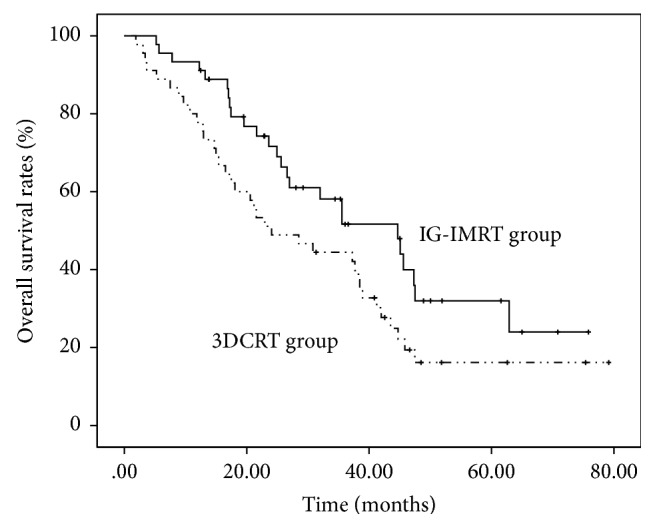
OS rates according to the modality of radiotherapy. Patients who received IG-IMRT showed longer median survival (44.7 versus 24.0 months, *P* = 0.046) than patients who received 3D-CRT.

Patients that received IG-IMRT showed significantly higher 1-year survival (93.3 versus 77.8%, *P* = 0.036) and 2-year survival (73.3 versus 51.1%, *P* = 0.030) and longer median survival (44.7 versus 24.0 months, *P* = 0.046) than patients that received 3D-CRT (Figure 3).

On univariate analysis, Child-Pugh classification (A versus B, *P* = 0.044), size of tumor (<8 versus ≥8 cm, *P* = 0.014), having received TACE before RT (yes versus no, *P* < 0.001), and RT modality (IG-IMRT versus 3D-CRT, *P* = 0.046) were significantly associated with OS. RT modality (*P* = 0.012), TACE before RT (*P* < 0.001), and size of tumor (*P* < 0.001) were significantly related to clinical prognosis by multivariate analysis of OS. The results of the univariate and multivariate analysis are summarized in Table 3.

### 3.5. Toxicity

No grade IV toxicity was observed in either group, and the common toxicity of radiotherapy was acute upper gastrointestinal (GI) toxicity, liver dysfunction, and hematological toxicity. GI toxicity includes anorexia, nausea, vomiting, and abdominal discomfort. The increase of Child-Pugh score was also evaluated as a toxicity index. There was no significant difference in the toxicity between the two groups (Table 4). No apparent radiation-induced liver disease was observed.

### 3.6. Failure Patterns

At the end of this study, 25 patients had died in the IG-IMRT group. 18 patients died of hepatic decompensation or tumor progression (or both), 3 patients died of multiple organ failure, 2 patients died of gastrointestinal hemorrhage, 1 patient died of lung metastasis, and 1 patient died of biliary tract infection. In the 3D-CRT group, 36 patients died. 22 patients died of hepatic decompensation or tumor progression (or both), 4 patients died of lung metastases, 4 patients died of multiple organ metastases, 2 patients died of gastrointestinal hemorrhage, 1 patient died of a ruptured liver cancer with hemorrhage, and the cause of death of 3 patients was undefined.

## 4. Discussion

As an advanced technique integrating IMRT and IGRT, helical tomotherapy is inherently capable of acquiring CT images of the patient in the treatment position and using this information for image guidance to offer an efficient, accurate, and safe treatment [16]. With the wide application of IG-IMRT, its advantages are also emerging in various tumor treatments [17–19]. In radiotherapy for HCC patients, IG-IMRT provides better uniformity for the PTV dose coverage than both IMRT and 3D-CRT [20]. A significant dosimetric gain and fewer tissue complications for patients with multiple tumors that underwent IG-IMRT in a shorter delivery time have also been reported [21].

In our study, the IG-IMRT group received a significantly higher dose (BED) and less fractions of RT than the 3D-CRT group. Regarding the whole liver, V5 was higher in IG-IMRT plans than 3D-CRT plans, while V10, V20, V30, and MDTNL showed no significant differences, which is consistent with previous reports [22]. Despite the increase in V5, IG-IMRT provided a significantly higher therapeutic dose in a shorter treatment period with similar and well-tolerated toxicity, which is a prerequisite for the hypofractioned radiotherapy [5].

The dose difference of IG-IMRT and 3DCRT was just less than 10% in BED (69.4 Gy versus 63.9 Gy). However, the treatment outcomes have bigger differences. ORRs were 57.3% versus 33.3%, DCRs were 95.6% versus 88.9%, 1- and 2-year overall survival rates were 93.3%/73.3% versus 77.8%/51.1%, and median overall survival was 44.7 months versus 24.0 months in the IG-IMRT and 3DCRT, respectively. There are some similar studies reporting that the higher therapeutic dose applied by IG-IMRT leads to excellent local control within the radiation field and a potential survival benefit [23, 24]. It is difficult to understand that the numerical difference of the doses between two groups is not obvious, but there is a big difference in effect. We should note that the comparison was made between two different radiotherapy modalities rather than different doses in the two groups. In radiotherapy, IG-IMRT performed the established radiotherapy plan more accurately than 3DCRT, which might lead to greater differences between the two groups in the actually delivered dose of the hepatic tumors. Furthermore, the effects of different radiotherapy modalities on the microenvironment are also different. As we know, there are many unknown areas of radiobiology for hepatocellular carcinoma. Other than direct damage to tumor, is it related to microenvironment changes of tumor and surrounding normal tissues caused by radiation? This is also worth our further exploration. The experimental research on this aspect has been carried out in our department. We are focused on irradiation facilitating Fas ligand secretion in hepatoma cells and increasing both hepatocytes and cancer cells injury [25]. In addition, different radiotherapy fractions were applied in the two groups. Further study on whether the fraction method would affect the treatment outcomes is also required.

After radiotherapy, some patients underwent curative therapies including tumor resection, liver transplantation, and radiofrequency ablation. As a conversion therapy or bridge therapy, whether IG-IMRT is superior to conventional radiotherapy requires studies with large sample.

Our results show that the IG-IMRT group had a significantly longer median survival than the 3D-CRT group, and the tumor size was also significantly associated with OS. We noticed that tumor size was the main factor limiting the prescription dose; tumor size determined the GTV, and the GTV determined the radiation volume. Higher prescription doses could not be applied due to the potential toxicity to OARs, resulting in reduced treatment efficacy. As an advanced radiotherapy technology, IG-IMRT eased this limitation to some extent. Furthermore, a role for the combination of TACE and RT in the treatment of HCC has been reported [26, 27], and we noticed that patients that received TACE before radiotherapy had more survival benefits compared with those that received TACE after radiotherapy on multivariate analysis of OS.

The safety and feasibility of IG-IMRT in liver cancer have been widely demonstrated [28, 29]. Here, the overall toxicity, including upper gastrointestinal (GI) toxicity, liver dysfunction, and hematological toxicity, was similar in both the IG-IMRT and 3D-CRT treatment groups. After radiotherapy, the gastroscopy is not a routine examination during follow-up, and therefore we cannot tell whether GI bleeding is esophageal and gastric varices bleeding or is associated with radiation. Patients with HCC suffer from portal hypertension due to cirrhosis or tumor thrombus, which results in esophageal and gastric varices bleeding. In our study, 4 patients died of gastrointestinal hemorrhage. All of the 4 patients had symptoms of haematemesis, which is one of the most typical manifestations of esophageal and gastric varices bleeding. Gastrointestinal tract was a high priority OAR in the process of formulating the radiotherapy plan, and we made sure that it was under tolerable dose. So we are inclined to think that the GI bleeding was not associated with the late complications or toxicity of RT.

With the continuous development of radiotherapy equipment, medical imaging, and computer technology, radiotherapy has become more important in the treatment of HCC. Although our study had several limitations, such as limited sample volume and a lack of random sampling, hypofractioned IG-IMRT provided a potential survival benefit and had satisfactory safety profile. However, the appropriate prescription dose and fraction of radiotherapy were not identified in this study, which will require additional randomized studies.

## 5. Conclusion

Compared with 3D-CRT, hypofractioned IG-IMRT provided a higher therapeutic dose in a shorter treatment period with similar and well-tolerated toxicity, which conferred a potential survival benefit.

## Figures and Tables

**Table 1:** Patient characteristics.

Characteristic	IG-IMRT (*n* = 45)	3D-CRT (*n* = 45)	*P* value
Gender			
Male	36	35	0.796
Female	9	10	
Age (years)			
<60	20	25	0.292
≥60	25	20	
KPS			
<90	13	5	0.035
≥90	32	40	
HBsAg			
Negative	10	7	0.419
Positive	35	38	
Total bilirubin (*µ*mol/L)			
≤34	39	41	0.502
>34	6	4	
Albumin (g/L)			
≤35	37	38	0.777
>35	8	7	
Child-Pugh classification			
A	40	42	0.459
B	5	3	
AFP (ng/mL)			
≤20	26	20	0.356
20–400	8	8	
≥400	11	17	
Tumor size (cm)			
≤8	35	33	0.624
>8	10	12	
Number of tumors			
Single	30	25	0.280
Multiple	15	20	
TACE (before RT)			
No	10	11	0.803
Yes	35	34	
TACE frequency			
0	10	11	0.537
1-2	24	19	
>2	11	15	

**Table 2:** Dose distribution.

Variables	IG-IMRT (*n* = 45)	3D-CRT (*n* = 45)	*P* value
RT dose (Gy)			
Average	53.70 ± 6.88	54.74 ± 5.56	0.427
BED average	69.38 ± 10.59	63.96 ± 6.44	0.004
RT fraction (Fx)	17.00 ± 5.35	27.18 ± 3.14	0.001
Dose to liver			
V5 (%)	71.20 ± 17.45	58.02 ± 17.69	0.001
V10 (%)	55.78 ± 19.60	50.09 ± 15.46	0.116
V20 (%)	35.96 ± 17.58	37.69 ± 12.25	0.595
V30 (%)	23.67 ± 14.71	28.58 ± 11.01	0.079
MDTNL^a^ (cGy)	1797.73 ± 728.36	1837.64 ± 548.34	0.768

^a^Mean dose to normal liver (MDTNL) was defined as the mean dose to the whole liver minus the GTV.

**Table 3:** Univariate and multivariate analysis of OS.

Variables	Median OS (months)	*P* value	
		Univariate	Multivariate
Gender			
Male	35.54 ± 5.08	0.347	
Female	22.95 ± 1.95		
Age (years)			
<60	38.26 ± 3.02	0.454	
≥60	26.59 ± 6.34		
KPS			
<90	38.46 ± 6.30	0.606	
≥90	30.82 ± 6.26		
HBsAg			
Negative	22.95 ± 5.58	0.476	
Positive	37.67 ± 3.90		
Total bilirubin (*µ*mol/L)			
≤34	37.25 ± 3.99	0.209	
>34	12.85 ± 4.43		
Albumin (g/L)			
≤35	28.49 ± 6.28	0.317	
>35	37.25 ± 5.85		
Child-Pugh classification			
A	37.25 ± 4.06	0.044	0.144
B	17.21 ± 6.50		
AFP (ng/mL)			
≤20	41.25 ± 10.70	0.247	
20–400	35.54 ± 5.29		
≥400	25.61 ± 4.40		
Tumor size (cm)			
≤8	38.26 ± 4.39	0.014	0.000
>8	19.54 ± 3.25		
Number of tumors			
Single	37.25 ± 6.94	0.216	
Multiple	35.54 ± 7.42		
TACE (before RT)			
No	14.98 ± 3.18	0.000	0.000
Yes	38.46 ± 3.95		
TACE (after RT)			
No	37.25 ± 7.12	0.269	
Yes	28.49 ± 7.68		
RT modality			
IG-IMRT	44.66 ± 7.78	0.046	0.012
3D-CRT	24.07 ± 6.47		

**Table 4:** Radiation toxicities.

Variables	Grade	IG-IMRT (*n* = 45)	3D-CRT (*n* = 45)	*P* value
GI toxicity	None	37	29	0.114
	I	6	9	
	II	2	7	
	III/IV	0	0	
Increase of alanine aminotransferase	None	39	37	0.496
	I	5	7	
	II	1	0	
	III	0	1	
	IV	0	0	
Increase of Aspartate aminotransferase	None	32	34	0.425
	I	13	9	
	II	0	1	
	III	0	1	
	IV	0	0	
Thrombocytopenia	None	28	23	0.434
	I	8	14	
	II	5	3	
	III	4	5	
	IV	0	0	
Decrease in hemoglobin	None	32	25	0.303
	I	10	16	
	II	3	4	
	III/IV	0	0	
Increase of Child-Pugh score	None	28	23	0.503
	1	8	14	
	2	5	3	
	3	4	5	
	Above 3	0	0	
